# Supplementation of Vitamin D and Mental Health in Adults with Respiratory System Diseases: A Systematic Review of Randomized Controlled Trials

**DOI:** 10.3390/nu15040971

**Published:** 2023-02-16

**Authors:** Dominika Głąbska, Aleksandra Kołota, Katarzyna Lachowicz, Dominika Skolmowska, Małgorzata Stachoń, Dominika Guzek

**Affiliations:** 1 Department of Dietetics, Institute of Human Nutrition Sciences, Warsaw University of Life Sciences (WULS-SGGW), 159C Nowoursynowska Street, 02-776 Warsaw, Poland; 2 Department of Food Market and Consumer Research, Institute of Human Nutrition Sciences, Warsaw University of Life Sciences (WULS-SGGW), 159C Nowoursynowska Street, 02-776 Warsaw, Poland

**Keywords:** tuberculosis of the respiratory system, respiratory tract infection, asthma, chronic obstructive pulmonary disease, vitamin D, supplementation of vitamin D, mental health, depression, anxiety, quality of life, randomized controlled trials (RCTs), systematic review

## Abstract

Vitamin D is indicated to be beneficial for the prevention and treatment of both respiratory health and mental health problems, while mental health issues are a common consequence of diseases of the respiratory system. The aim of the presented systematic review was to gather available evidence regarding the influence of the supplementation of vitamin D on mental health in adults with respiratory system diseases obtained within randomized controlled trials (RCTs). The systematic review was conducted on the basis of the PubMed and Web of Science databases in agreement with the guidelines of Preferred Reporting Items for Systematic Reviews and Meta-Analyses (PRISMA), while being registered within the database of the International Prospective Register of Systematic Reviews (PROSPERO) (CRD42020155779). A total of 8514 studies published before September 2021 were screened and 5 RCTs were included, which were assessed using the revised Cochrane risk-of-bias tool for randomized trials. Screening, inclusion, reporting, and assessment were conducted by two researchers independently. The studies focused on the assessment of patients with chronic obstructive pulmonary disease, but also increased susceptibility to respiratory tract infections, pulmonary tuberculosis, and bronchial asthma. The studies were conducted for various periods of time—from 2 months to a year—while the dose of vitamin D applied was also diverse—from 4000 IU applied daily, to 100,000 IU applied weekly, or monthly. The psychological measures applied within the studies allowed the assessment, mainly, of quality of life, but also well-being, and depression. For the majority of studies, some concerns regarding risk of bias were defined, resulting from the randomization process and selection of reported results; however, for one study, the risk was even defined as high. Within the included studies, three studies confirmed a beneficial effect of vitamin D (including those with a high risk of bias), but two studies did not confirm it. Taking into account the evidence gathered, in spite of a positive influence of vitamin D on mental health in individuals with increased susceptibility to respiratory tract infections and bronchial asthma, the conducted systematic review is not a strong confirmation of the beneficial effect of the supplementation of vitamin D on mental health in adults with respiratory system diseases.

## 1. Introduction

The diseases of the respiratory system are a large group of various diseases with a diverse etiology, including infections, toxic agents, accidents, risky behaviors such as smoking, and genetic factors [1]. They are classified within the International Classification of Diseases (ICD-11), in the 12th chapter, but tuberculosis of the respiratory system, classified within infectious and parasitic diseases (1st chapter), may be also considered [2]. Within this group of various diseases and conditions, they may be divided based on the pathology and transmission, which also influence diagnosis and treatment [3], into groups of infectious diseases (for example, pneumonia or tuberculosis of the respiratory system) and noncommunicable diseases (for example, asthma, chronic obstructive pulmonary disease, cystic fibrosis, or lung cancer) [4].

As the diseases of the respiratory system may be associated with increased fatigue and dyspnea, they may, as a result, reduce the possibility of habitual activities and work performance, resulting in reduced general quality of life [5]. Moreover, as diseases of the respiratory system result in symptoms including breathlessness, chronic fatigue, and cough, which in some diseases of the respiratory system can only be alleviated but not removed, the need for a psychological approach is emphasized for the well-being of patients [6].

Not only is the quality of life in diseases of the respiratory system deprived, but also other mental health problems, as a result of the underlying disease, are common and chronic. At the same time, these mental health problems influence worse outcomes of the underlying disease [7]. Considering this fact, improving mental health diagnostics and including psychiatric care may improve not only mental health, but the results of diseases of the respiratory system as well [8].

Except for the other methods of psychological treatment and drugs, which must be applied if needed [9], recent studies indicate that for mental health problems, dietary interventions may be also applied as supportive therapies which allow the reduction of mental health symptoms [10]. Interestingly, for diseases of the respiratory system, dietary modifications are also indicated as potential preventive and therapeutic factors, as they may influence the development, progression, and treatment of the diseases [11].

Vitamin D is indicated among such nutritional factors which are defined as beneficial both for the treatment and prevention of respiratory diseases [12] and mental health problems [13]. Its deficiency may lead to an increased risk of asthma and wheezing diseases, but also of depression and schizophrenia [14]. This results from vitamin D being engaged in immunomodulation, while its receptors are expressed by a majority of immune cells, so this nutrient is indicated to be a potentially important factor in the prevention and therapy of numerous diseases [15].

Based on the presented background, the aim of the presented systematic review was to gather available evidence regarding the influence of the supplementation of vitamin D on mental health in adults with respiratory system diseases obtained within randomized controlled trials (RCTs).

## 2. Materials and Methods

### 2.1. The Systematic Review Design and Registration

The systematic review was conducted in agreement with the guidelines of Preferred Reporting Items for Systematic Review and Meta-Analyses (PRISMA) [16] and registered in the International Prospective Register of Systematic Reviews (PROSPERO) (CRD42020155779).

The studies published until September 2021 and available within PubMed and/or Web of Science databases were screened to include RCTs and assess them using revised Cochrane risk-of-bias tool for randomized trials. As the coronavirus disease 2019 (COVID-19) may influence symptoms in the respiratory system [17], as well as the global pandemic having a major impact on mental health, based on psychological distress [18], the systematic literature search was divided into two stages—conducted before October 2019 and conducted since October 2019—while for the second stage, the data extraction was planned to additionally include any information about COVID-19 incidence within the studied group, if available.

The applied procedure was based on that previously applied for the assessment of the association between vitamin D and mental health in children [19] and adults [20], as well as for specific populations of patients diagnosed with diabetes [21], multiple sclerosis [22], as well as inflammatory bowel diseases and irritable bowel syndrome [23]. However, respiratory system diseases are now the key focus for the gathered studies.

### 2.2. The Search Strategy and Eligibility Assessment

An electronic search was aimed at gathering RCTs regarding the influence of the supplementation of vitamin D on mental health in adults with respiratory system diseases, based on the inclusion criteria listed:

- studied an adult population;
- studied a population with any diagnosed respiratory system disease, based on ICD-11: all included in the 12th chapter, and tuberculosis of the respiratory system (1B10) [2];
- applied oral supplementation of a specified dose of vitamin D and compared with placebo;
- any mental health outcome monitored within the study using a valid psychological measure (either subjective or objective);
- study defined as RCT;
- study available in a peer-reviewed journal.
- The exclusion criteria were applied as listed:
- animal model study;
- influence of a combination of multiple nutrients presented;
- studied a population of pregnant women;
- studied a population of patients with concurrent eating disorders;
- studied a population of patients with concurrent intellectual disabilities;
- studied a population of patients with concurrent neurological disorders;
- study not published in English.

The population, intervention/exposure, comparator, outcome, and study design (PICOS) criteria for the presented study are described in Table 1.

### 2.3. The Searching Procedure and Data Extraction

The separate detailed search strategy for PubMed and Web of Science databases is presented in Supplementary Table S1.

After searching, duplicates were removed manually. Two researchers then reviewed the relevance of the titles of articles, abstracts of articles (for records included based on titles), and the full texts of articles (for records included based on abstracts), based on previously developed inclusion criteria and exclusion criteria. In order to obtain a full text of the study, electronic databases and libraries were searched, and if not available, the corresponding authors were contacted and asked for them. Any disagreement was discussed with a third investigator until consensus was achieved.

**Figure 1:**
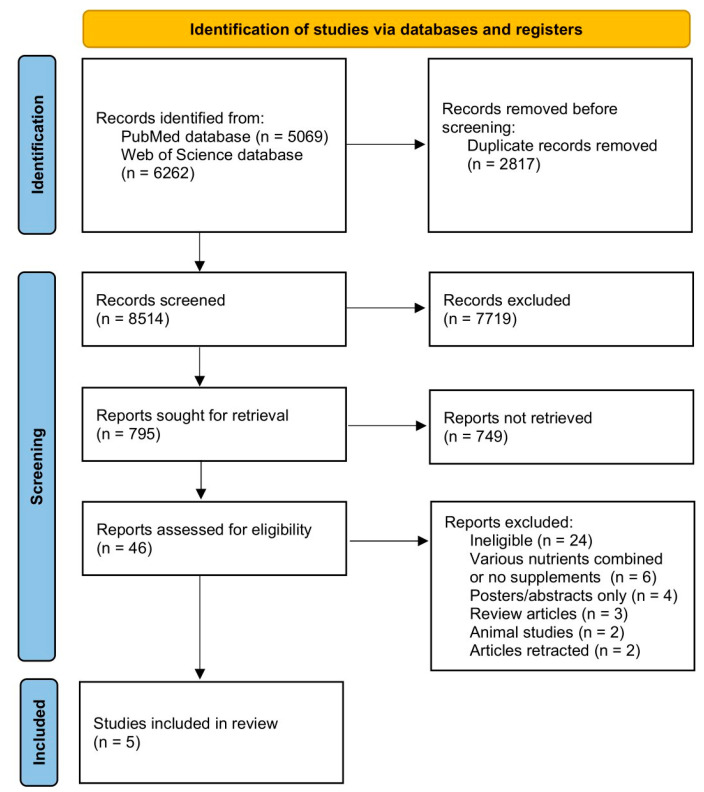
The procedure of identifying, selecting, assessing eligibility, and including studies.

The procedure of identifying, selecting, assessing eligibility, and including studies is presented in Figure 1.

Once the eligible studies were included, they were analyzed to derive the data needed to describe the study and the influence of the supplementation of vitamin D on mental health in adults with respiratory system diseases. The general description of the study included: authors and year of publication, country and detailed location, general description of the studied population, as well as the period of study. The description of the studied population included the number, gender and age of participants, as well as inclusion and exclusion criteria. The description of the supplementation of vitamin D included the dosage regimen, intervention duration, and time of intervention, while the description of the assessment of mental health included an applied psychological measure. The description of the prominent observations and conclusions was based on those drawn up by authors.

If possible, all data were obtained from a published study. If this was not possible, other publications referred to within the study were addressed. If this was not possible, the corresponding authors were contacted and asked for them. Two researchers independently extracted the data, but if any disagreement appeared, it was discussed with a third investigator until consensus was achieved.

### 2.4. The Quality of Studies and Risk-of-Bias Assessment

The quality of studies was determined based on a risk of bias defined for the studies [24]. The revised Cochrane risk-of-bias tool for randomized trials with the RoB 2 tool (7.0) [25] was applied for the assessment of the risk of bias, as it is the most frequently used for randomized trials [26].

The revised Cochrane risk-of-bias tool for randomized trials consists of an assessment of five distinct domains of the risk of bias: (1) arising from the randomization process; (2) due to deviations from the intended interventions; (3) due to missing outcome data; (4) in the measurement of the outcome; and (5) in the selection of the reported results. Afterwards, it was assessed for the overall risk [27]. The risk-of-bias assessment within the revised Cochrane risk-of-bias tool for randomized trials for each domain is formulated as: (1) low risk of bias; (2) some concerns; or (3) high risk of bias, while the final assessment is based on the summarized assessment [26].

Two researchers independently assessed the studies, but if any disagreement appeared, it was discussed with a third investigator until consensus was achieved.

## 3. Results

The general descriptions of the studies included in the systematic review [28,29,30,31,32] are presented in Table 2. The studies focused on the assessment of patients with chronic obstructive pulmonary disease (COPD) [28,31], but also increased susceptibility to respiratory tract infections [29], pulmonary tuberculosis [30], and bronchial asthma [32]. The majority of studies were conducted in European countries: Belgium [28], Sweden [29], and Spain [32], but also in China [30] and Iran [31].

The descriptions of the studied populations within the studies included in the systematic review are presented in Table 3. The studies were conducted mostly in quite large samples of more than 100 participants (studied group and placebo group combined), with various proportions of female and male participants [28,29,30,32], but one study was conducted in a medium-sized sample of fewer than 100 participants, with a very small share of women [31]. The studies were conducted in populations of middle-aged adults of various ages—from a population of individuals in their 30–40s [30], or a population of individuals in their 50s [28,32], to a population of individuals in their 60s [28,31]. The inclusion and exclusion criteria were developed based on the studied population (studied disease of the respiratory system), but with additional criteria in order to gather a sample with a major depressive disorder diagnosed [30], or vitamin D deficiency [31,32].

The descriptions of the supplementation of vitamin D, accompanied by the descriptions of the assessments of mental health within the studies included in the systematic review are presented in Table 4. The studies were conducted for various periods of time—2 months [30], 6 months [31,32], or a year [28,29]. The dose of vitamin D applied within the studies was also diverse— 4000 IU applied daily [29], 16,000 IU applied weekly [32], 50,000 IU applied weekly or monthly [31], or 100,000 IU applied weekly [30] or monthly [28]. The psychological measures applied within the studies allowed the assessment, mainly, of quality of life [28,31,32], but also well-being [29], and depression [30].

The risk-of-bias assessments for studies, conducted using the revised Cochrane risk-of-bias tool for randomized trials, accompanied by the main results of the studies included in the systematic review, are presented in Table 5. For the majority of studies, some concerns were defined [28,30,31,32], resulting from a risk of bias arising from the randomization process [31,32], and from a risk of bias in selection of the reported results [28,30,31,32]. However, for one study [29], the risk was even defined as high. At the same time, it should be indicated that within the studies, three studies [29,31,32] confirmed the beneficial effect of vitamin D (including those with a high risk of bias [29]), but two studies did not confirm it [28,30].

## 4. Discussion

This systematic review aimed to gather available evidence regarding the influence of the supplementation of vitamin D on mental health in adults with respiratory system diseases obtained within randomized controlled trials (RCTs). However, the results should not be considered as a strong confirmation of a general positive effect of the supplementation of vitamin D on mental health in adults with respiratory system diseases, as the beneficial effect was observed for increased susceptibility to respiratory tract infections [29] and bronchial asthma [32], as well as for one study conducted for COPD [31], while for the other study conducted for COPD [28], and for pulmonary tuberculosis [30], such a beneficial effect was not observed. Not only should a general positive influence not be concluded, but also a beneficial effect in specific conditions cannot be stated, as only single RCTs confirming were gathered for each mentioned disease. However, an increased susceptibility to respiratory tract infections, bronchial asthma and COPD may be indicated as a promising area to be studied in the future.

The effects observed for increased susceptibility to respiratory tract infections are associated with the general influence of vitamin D, being associated with immune functions [15]. It is observed that it influences the immunity by triggering the induction of cathelicidin, being an antimicrobial peptide capable of mediating antimicrobial activity [33]. At the same time, vitamin D influences autophagy, as well as adaptive immune responses, by promoting regulatory lymphocytes [34]. It was confirmed by the systematic review and meta-analysis of RCTs by Bergman et al. [35], which indicated that vitamin D has a protective effect against respiratory tract infections. At the same time, the systematic review by Charan et al. [36] indicated that it may be even more observable in children than in adults. Some similar observations were formulated for hospital-acquired infections, including inter alia wound infections and sepsis [37], but vitamin D failed to be effective against respiratory infections after lung transplants [38]. During the COVID-19 pandemic, numerous studies also verified the effectiveness of vitamin D in the prevention and treatment of COVID-19 infection. Within the systematic review by Jordan et al. [39], it was concluded that its supplementation may play an important role in protecting from acute infections, and in the treatment of high-risk individuals, it may prevent progression to a critical clinical condition, and as a result, it may reduce mortality. Taking this into account, the supplementation of vitamin D is concluded to be safe option to prevent against acute respiratory tract infections [40].

For the effect of vitamin D on bronchial asthma, some conflicting data were obtained from clinical trials, but it is emphasized that vitamin D deficiency may influence the inflammatory response in the airways [41]. However, the effect of supplementation is not always observed [42]. Positive conclusions were formulated within a RCT by Arshi et al. [43], as they proved that the supplementation of vitamin D in patients, including adults and adolescents with mild to moderate persistent asthma, significantly improved forced expiratory volume in 1 s (FEV1). It is generally associated with low vitamin D levels in asthmatic patients, which may be improved during supplementation, and as a result, it may enhance asthma control [44], as the vitamin D status in asthmatic patients is associated with their lung function [45]. However, other studies did not provide such positive observations, as in children with mild asthma, no effect of supplementation of vitamin D on airway reactivity and inflammation was stated [46]. Taking this into account, it is indicated that there is some potential to use vitamin D in the prevention and treatment of asthma [47].

For COPD, similarly to respiratory tract infections and asthma, there are some beneficial observations. The RCT by Khan et al. [48] indicated that the supplementation of vitamin D in COPD patients may be effective in reducing the number of acute exacerbations. At the same time, the systematic review and meta-analysis by Zhu et al. [49] indicated that vitamin D status is inversely associated with the risk and severity of COPD, as well as with its exacerbations. Similarly, the systematic review and meta-analysis of individual participant data from RCTs by Jolliffe et al. [50] indicated that the supplementation of vitamin D may effectively reduce the rate of COPD exacerbations in patients with low baseline vitamin D levels, but not in those with higher ones. Taking this into account, the routine control of vitamin D status is suggested to be undertaken in COPD patients [51].

The results of the studies described above confirm or at least suggest the positive role of vitamin D in respiratory functions. At the same time, the beneficial role of vitamin D in mental health in the general population is known, which was observed mainly for depression [52,53,54], but also for the occurrence of negative emotions [55], and for quality of life [56]. The mechanism of the influence of vitamin D on mental health is associated with the fact that vitamin D may cross the blood–brain barrier, which results in the activation of receptors in brain cells and a direct impact in the central nervous system [57]. At the same time, it is suggested that vitamin D and Vitamin D receptors (VDRs) may influence the regulation of human behavior, as VDRs are present in the cortex, cerebellum, and limbic system of the brain [58]. Notwithstanding this, the mechanism is not simple, due to the fact that VDR genes are polymorphic with frequent variations, causing vitamin-D-related dysfunctions [59].

However, the question about the potential effect of vitamin D on mental health in patients with respiratory system diseases is still unanswered. At the same time, concurrent diseases and other disorders may interfere, as they may be also associated with the role of vitamin D, as indicated for obesity [60], or even the common cold [61]. In spite of the fact that the number of studies conducted for patients with respiratory system diseases indicated some positive observations, it was not sufficient to formulate explicit conclusions.

## 5. Conclusions

Taking into account the evidence gathered, in spite of a positive influence of vitamin D on mental health in individuals with an increased susceptibility to respiratory tract infections and bronchial asthma, the conducted systematic review is not a strong confirmation of the beneficial effect of supplementation of vitamin D on mental health in adults with respiratory system diseases.

## Figures and Tables

**Table 1:** The population, intervention/exposure, comparator, outcome, and study design (PICOS) criteria.

PICOS Criterion	Inclusion	Exclusion
Population	Adult patients with any respiratory system disease diagnosed	Pregnant women, patients with any concurrent eating disorders, patients with any concurrent intellectual disabilities, patients with concurrent neurological disorders
Intervention/exposure	Vitamin D oral supplementation of known dose applied	Multiple nutrient supplementation applied
Comparison	Studied group compared with control group without vitamin D supplementation	No comparison with placebo group
Outcome	Any mental health outcome monitored	No valid mental health outcome measure applied
Study design	Randomized controlled trial (RCT) published as article in peer-reviewed journal	Studies not published in English, animal model studies

**Table 2:** The general descriptions of the studies included in the systematic review.

Ref.	Authors and Year of the Study	Country and Detailed Location	General Description of the Studied Population	Period of the Study
[28]	Lehouck et al., 2012	Belgium, Leuven	Patients with moderate to very severe chronic obstructive pulmonary disease and a history of recent exacerbations screened at the University Hospitals Leuven	January 2008–April 2010 *
[29]	Bergman et al. 2015	Sweden, Huddinge	Patients with an increased susceptibility to respiratory tract infections from the Immunodeficiency Unit, Karolinska University Hospital, Huddinge, Sweden	March 2010–June 2011 *
[30]	Zhang et al. 2018	China, Southeast China	Patients with pulmonary tuberculosis and major depressive disorder from four hospitals	July 2015–September 2017
[31]	Alavi Foumani et al. 2019	Iran, Rasht	Patients with chronic obstructive pulmonary disease referred to the respiratory clinic of Razi Hospital	August 2015–June 2016
[32]	Andújar-Espinosa et al. 2021	Spain, Murcia	Patients with bronchial asthma with vitamin D deficiency hospitalized or consulted in the emergency department at Morales Meseguer Hospital	June 2016–August 2017

* data provided on request.

**Table 3:** The descriptions of the studied populations within the studies included in the systematic review.

Ref.	Number of Participants (Female)	Age (Mean/Mean with SD)	Inclusion and Exclusion Criteria
[28]	182 (37)	For vitamin D group: 68 ± 9 years For placebo group: 68 ± 8 years	Inclusion: >50 years; current or former smokers; moderate to very severe chronic obstructive pulmonary disease (COPD) according to the Global Initiative for Chronic Obstructive Lung Disease (GOLD) definition (postbronchodilator FEV1–FVC ratio <0.7) and FEV1 less than 80% predicted; history of recent exacerbations Exclusion: history of hypercalcemia, sarcoidosis, or active cancer; treatment with vitamin D supplements (newly discovered symptomatic osteoporosis); long-term azithromycin treatment, with antibacterial and anti-inflammatory functions
[29]	124 (91)	For vitamin D group: 55.4 years For placebo group: 50.8 years	Inclusion: aged 18–75 years; increased susceptibility to respiratory tract infections (>42 days with symptoms from the respiratory tract during a 12 months) Exclusion: prophylactic treatment with antibiotics; history of hypercalcemia or stones in the urinary tract; sarcoidosis; supplementation with vitamin D_3_ > 400 IU/day; HIV-infection; pregnancy
[30]	123 (20)	For vitamin D group: 38.3 ± 12.4 years For placebo group: 40.2 ± 11.9 years	Inclusion: age ≥ 18 years; recurrent pulmonary tuberculosis (PTB) (relapse of original episode or an exogenous reinfection caused by a different strain of Mycobacterium tuberculosis); major depressive disorder (Diagnostic and Statistical Manual of Mental Disorders-IV criteria); standard tuberculosis re-treatment Exclusion: preexisting renal or hepatic failure; pulmonary silicosis; malignancy; metastatic malignant disease; sarcoidosis; hyperparathyroidism; nephrolithiasis; HIV; active diarrhea; hypercalcemia; pregnancy; lactation; steroid, cytotoxic drug treatment or other immunosuppressant therapies in the month before; intolerance of vitamin D or first-line anti-tuberculosis therapies; cognitive deficits; illiteracy or inability to answer the questionnaire; severe depressive symptoms before treatment
[31]	63 (3)	For vitamin D group: 67.9 ± 7.9 years For placebo group: 68.4 ± 7.8 years	Inclusion: chronic obstructive pulmonary disease (COPD); 25 (OH)vitamin D levels in blood of 10–30 ng/mL Exclusion: congestive heart failure; osteoporosis; acute myocardial infarction; glomerular filtration rate ≤ 45 mL/min/1.73 m^2^; hypercalcemia; malignancy; sarcoidosis; long-term azithromycin use; taking antiepileptic drugs
[32]	112 (87)	For vitamin D group: 54.6 ± 15.8 years For placebo group: 56.6 ± 15.0 years	Inclusion: >18 years old; bronchial asthma as a primary or secondary diagnosis; 25(OH)vitamin D levels in blood of <30 ng/mL Exclusion: smoking > 10 packs a year; current use of vitamin D supplements; kidney disease (serum creatinine > 2 mg/dL); hypercalcemia (serum calcium corrected with proteins > 10.5 mg/dL); history of recurrent kidney stones (≥3 episodes); pathologies affecting intestinal vitamin D absorption; pregnancy; breastfeeding; severe psychosocial problems (such as dementia, alcoholism or other drug addictions, or psychiatric disorders such as major active depression or schizophrenia)

FEV1–FVC ratio—Tiffeneau-Pinelli index; FEV1—forced expiratory volume in 1 second; FVC—full, forced vital capacity.

**Table 4:** The descriptions of the supplementation of vitamin D, accompanied by the descriptions of the assessments of mental health within the studies included in the systematic review.

Ref.	Vitamin D Supplementation Dose Regimen		Vitamin D Supplementation Duration	Psychological Measure of Depression
	Dose	Time		
[28]	100,000 IU	Monthly	1 year	Quality of life assessed by the Chronic Respiratory Questionnaire (CRQ)
[29]	4000 IU	Daily	1 year	Single-item assessment of well-being during the study (“How do you rate your overall health during the study year compared with the year before inclusion?”)
[30]	100,000 IU	Weekly	8 weeks	Chinese version of Beck Depression Inventory (BDI) II
[31]	50,000 IU	Weekly for 2 months + monthly for 4 months	6 months (total)	Quality of life assessed using the Chronic Obstructive Pulmonary Disease (COPD) Assessment Test (CAT)
[32]	16,000 IU	Weekly	6 months	Mini-Asthma Quality of Life Questionnaire (AQLQ)

**Table 5:** The risk of bias assessments for studies, conducted using the revised Cochrane risk-of-bias tool for randomized trials, accompanied by the main results of the studies included in the systematic review.

Ref.	D1	D2	D3	D4	D5	Overall Bias	Main Result of the Study *
[28]	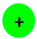	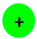	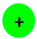	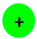	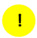	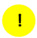	Not confirming
[29]	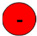	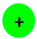	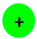	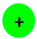	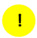	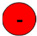	Confirming
[30]	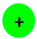	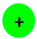	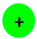	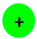	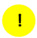	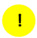	Not confirming
[31]	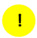	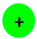	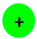	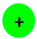	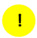	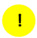	Confirming
[32]	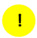	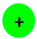	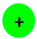	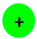	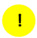	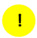	Confirming

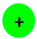
—Low risk; 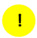
—Some concerns 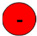
—High risk; * the summary of conclusions defined as confirming (if confirming a positive influence of vitamin D supplementation on mental health) or not confirming (if not confirming a positive influence of vitamin D supplementation on mental health); domains of risk of bias: D1—arising from the randomization process; D2—due to deviations from the intended interventions; D3—due to missing outcome data; D4—in measurement of the outcome; D5—in the selection of the reported results.

## Supplementary Materials

The following are available online at https://www.mdpi.com/article/10.3390/nu15040971/s1. Supplementary Table S1. The detailed electronic search strategy applied within the systematic review separately for PubMed and Web of Science databases. Supplementary Table S2. Descriptions of the studies and studied populations within studies included in a systematic review.

## References

[1] National Center for Biotechnology Information (US) (1998). Genes and Disease.

[2] ICD-11 for Mortality and Morbidity Statistics. https://icd.who.int/browse11/l-m/en.

[3] Shukla S.D., Swaroop Vanka K., Chavelier A., Shastri M.D., Tambuwala M.M., Bakshi H.A., Pabreja K., Mahmood M.Q., O’Toole R.F. (2020). Chronic respiratory diseases: An introduction and need for novel drug delivery approaches. Targeting Chronic Inflammatory Lung Diseases Using Advanced Drug Delivery Systems.

[4] Vos T., Barber R.M., Bell B., Bertozzi-Villa A., Biryukov S. (2015). Global, regional, and national incidence, prevalence, and years lived with disability for 301 acute and chronic diseases and injuries in 188 countries, 1990–2013: A systematic analysis for the Global Burden of Disease Study 2013. Lancet.

[5] Cappa V., Marcon A., Di Gennaro G., Chamitava L., Cazzoletti L., Bombieri C., Nicolis M., Perbellini L., Sembeni S., de Marco R. (2019). Health-related quality of life varies in different respiratory disorders: A multi-case control population based study. BMC Pulm. Med..

[6] Booth S., Johnson M.J. (2019). Improving the quality of life of people with advanced respiratory disease and severe breathlessness. Breathe.

[7] Hunter R., Barson E., Willis K., Smallwood N. (2021). Mental health illness in chronic respiratory disease is associated with worse respiratory health and low engagement with non-pharmacological psychological interventions. Intern. Med. J..

[8] Yohannes A.M., Newman M., Kunik M.E. (2019). Psychiatric Collaborative Care for Patients With Respiratory Disease. Chest..

[9] Cuijpers P., Quero S., Dowrick C., Arroll B. (2019). Psychological Treatment of Depression in Primary Care: Recent Developments. Curr. Psychiatry Rep..

[10] Firth J., Marx W., Dash S., Carney R., Teasdale S.B., Solmi M., Stubbs B., Schuch F.B., Carvalho A.F., Jacka F. (2019). The Effects of Dietary Improvement on Symptoms of Depression and Anxiety: A Meta-Analysis of Randomized Controlled Trials. Psychosom. Med..

[11] Berthon B.S., Wood L.G. (2015). Nutrition and respiratory health--feature review. Nutrients.

[12] Hughes D.A., Norton R. (2009). Vitamin D and respiratory health. Clin. Exp. Immunol..

[13] Dursun S. (2010). Vitamin D for mental health and cognition. CMAJ.

[14] Holick M.F., Chen T.C. (2008). Vitamin D deficiency: A worldwide problem with health consequences. Am. J. Clin. Nutr..

[15] Sassi F., Tamone C., D’Amelio P. (2018). Vitamin D: Nutrient, Hormone, and Immunomodulator. Nutrients.

[16] Moher D., Liberati A., Tetzlaff J., Altman D.G. (2009). PRISMA Group. Preferred reporting items for systematic reviews and meta-analyses: The PRISMA statement. PLoS Med..

[17] Brosnahan S.B., Jonkman A.H., Kugler M.C., Munger J.S., Kaufman D.A. (2020). COVID-19 and Respiratory System Disorders: Current Knowledge, Future Clinical and Translational Research Questions. Arterioscler. Thromb. Vasc. Biol..

[18] Xiong J., Lipsitz O., Nasri F., Lui L.M.W., Gill H., Phan L., Chen-Li D., Iacobucci M., Ho R., Majeed A. (2020). Impact of COVID-19 pandemic on mental health in the general population: A systematic review. J. Affect. Disord..

[19] Głąbska D., Kołota A., Lachowicz K., Skolmowska D., Stachoń M., Guzek D. (2021). The Influence of Vitamin D Intake and Status on Mental Health in Children: A Systematic Review. Nutrients.

[20] Guzek D., Kołota A., Lachowicz K., Skolmowska D., Stachoń M., Głąbska D. (2021). Association between Vitamin D Supplementation and Mental Health in Healthy Adults: A Systematic Review. J. Clin. Med..

[21] Guzek D., Kołota A., Lachowicz K., Skolmowska D., Stachoń M., Głąbska D. (2021). Influence of Vitamin D Supplementation on Mental Health in Diabetic Patients: A Systematic Review. Nutrients.

[22] Głąbska D., Kołota A., Lachowicz K., Skolmowska D., Stachoń M., Guzek D. (2021). Vitamin D Supplementation and Mental Health in Multiple Sclerosis Patients: A Systematic Review. Nutrients.

[23] Głąbska D., Kołota A., Lachowicz K., Skolmowska D., Stachoń M., Guzek D. (2021). Vitamin D Supplementation and Mental Health in Inflammatory Bowel Diseases and Irritable Bowel Syndrome Patients: A Systematic Review. Nutrients.

[24] Assessing Risk of Bias in Non-Randomized Studies. Chapter 13.5.2.3. http://handbook-5-1.cochrane.org/.

[25] RoB 2: A Revised Cochrane Risk-of-Bias Tool for Randomized Trials. https://methods.cochrane.org/bias/resources/rob-2-revised-cochrane-risk-bias-tool-randomized-trials.

[26] Sterne J.A.C., Savović J., Page M.J., Elbers R.G., Blencowe N.S., Boutron I., Cates C.J., Cheng H.Y., Corbett M.S., Eldridge S.M. (2019). RoB 2: A revised tool for assessing risk of bias in randomised trials. BMJ.

[27] Minozzi S., Cinquini M., Gianola S., Gonzalez-Lorenzo M., Banzi R. (2020). The revised Cochrane risk of bias tool for randomized trials (RoB 2) showed low interrater reliability and challenges in its application. J. Clin. Epidemiol..

[28] Lehouck A., Mathieu C., Carremans C., Baeke F., Verhaegen J., Van Eldere J., Decallonne B., Bouillon R., Decramer M., Janssens W. (2012). High doses of vitamin D to reduce exacerbations in chronic obstructive pulmonary disease: A randomized trial. Ann. Intern. Med..

[29] Bergman P., Norlin A.C., Hansen S., Björkhem-Bergman L. (2015). Vitamin D supplementation to patients with frequent respiratory tract infections: A post hoc analysis of a randomized and placebo-controlled trial. BMC Res. Notes.

[30] Zhang L., Wang S., Zhu Y., Yang T. (2018). Vitamin D3 as adjunctive therapy in the treatment of depression in tuberculosis patients: A short-term pilot randomized double-blind controlled study. Neuropsychiatr. Dis. Treat..

[31] Alavi Foumani A., Mehrdad M., Jafarinezhad A., Nokani K., Jafari A. (2019). Impact of vitamin D on spirometry findings and quality of life in patients with chronic obstructive pulmonary disease: A randomized, double-blinded, placebo-controlled clinical trial. Int. J. Chron. Obstruct. Pulmon. Dis..

[32] Andújar-Espinosa R., Aparicio-Vicente M., Ruiz-López F.J., Salinero-González L. (2021). Influence of vitamin D supplementation on the quality of life of asthma patients: Findings from ACVID randomised clinical trial. Respir. Med..

[33] Liu P.T., Stenger S., Li H., Wenzel L., Tan B.H., Krutzik S.R., Ochoa M.T., Schauber J., Wu K., Meinken C. (2006). Toll-like receptor triggering of a vitamin D-mediated human antimicrobial response. Science.

[34] Pfeffer P.E., Hawrylowicz C.M. (2012). Vitamin D and lung disease. Thorax.

[35] Bergman P., Lindh A.U., Björkhem-Bergman L., Lindh J.D. (2013). Vitamin D and Respiratory Tract Infections: A Systematic Review and Meta-Analysis of Randomized Controlled Trials. PLoS One.

[36] Charan J., Goyal J.P., Saxena D., Yadav P. (2012). Vitamin D for prevention of respiratory tract infections: A systematic review and meta-analysis. J. Pharmacol. Pharmacother..

[37] Youssef D.A., Ranasinghe T., Grant W.B., Peiris A.N. (2012). Vitamin D’s potential to reduce the risk of hospital-acquired infections. Dermato-endocrinologyc.

[38] Vos R., Ruttens D., Verleden S.E., Vandermeulen E., Bellon H., Van Herck A., Sacreas A., Heigl T., Schaevers V., Van Raemdonck D.E. (2017). High-dose vitamin D after lung transplantation: A randomized trial. J. Heart Lung Transplant..

[39] Jordan T., Siuka D., Rotovnik N.K., Pfeifer M. (2022). COVID-19 and Vitamin D- a Systematic Review. Zdr. Varst..

[40] Martineau A.R., Jolliffe D.A., Hooper R.L., Greenberg L., Aloia J.F., Bergman P., Dubnov-Raz G., Esposito S., Ganmaa D., Ginde A.A. (2017). Vitamin D supplementation to prevent acute respiratory tract infections: Systematic review and meta-analysis of individual participant data. BMJ.

[41] Hall S.C., Agrawal D.K. (2017). Vitamin D and Bronchial Asthma: An Overview of Data From the Past 5 Years. Clin. Ther..

[42] Szymczak I., Pawliczak R. (2018). Can vitamin D help in achieving asthma control? Vitamin D “evisited’’: An updated insight. Adv. Respir. Med..

[43] Arshi S., Fallahpour M., Nabavi M., Bemanian M.H., Javad-Mousavi S.A., Nojomi M., Esmaeilzadeh H., Molatefi R., Rekabi M., Jalali F. (2014). The effects of vitamin D supplementation on airway functions in mild to moderate persistent asthma. Ann. Allergy Asthma Immunol..

[44] Menon B., Nima G., Dogra N., Mittal A., Kaur C., Mittal U. (2014). Evaluation of vitamin D in bronchial asthma and the effect of vitamin D supplementation on asthma severity and control: A randomised control trial. Eur. Respir J..

[45] Nasiri Kalmarzi R., Zamani A., Fathallahpour A., Ghaderi E., Rahehagh R., Kooti W. (2016). The relationship between serum levels of vitamin D with asthma and its symptom severity: A case-control study. Allergol. Immunopathol..

[46] Bar Yoseph R., Livnat G., Schnapp Z., Hakim F., Dabbah H., Goldbart A., Bentur L. (2015). The effect of vitamin D on airway reactivity and inflammation in asthmatic children: A double-blind placebo-controlled trial. Pediatr. Pulmonol..

[47] Lewis E., Casale T. (2011). Role of vitamin D in asthma. Therapy.

[48] Khan D.M., Ullah A., Randhawa F.A., Iqtadar S., Butt N.F., Waheed K. (2017). Role of Vitamin D in reducing number of acute exacerbations in Chronic Obstructive Pulmonary Disease (COPD) patients. Pak. J. Med. Sci..

[49] Zhu M., Wang T., Wang C., Ji Y. (2016). The association between vitamin D and COPD risk, severity, and exacerbation: An updated systematic review and meta-analysis. Int. J. Chron. Obstruct. Pulmon. Dis..

[50] Jolliffe D.A., Greenberg L., Hooper R.L., Mathyssen C., Rafiq R., de Jongh R.T., Camargo C.A., Griffiths C.J., Janssens W., Martineau A.R. (2019). Vitamin D to prevent exacerbations of COPD: Systematic review and meta-analysis of individual participant data from randomised controlled trials. Thorax.

[51] Mishra N.K., Mishra J.K., Srivastava G.N., Shah D., Rehman M., Latheef N.A., Maurya A., Rajak B.K. (2019). Should vitamin D be routinely checked for all chronic obstructive pulmonary disease patients?. Lung India..

[52] Vellekkatt F., Menon V. (2019). Efficacy of vitamin D supplementation in major depression: A meta-analysis of randomized controlled trials. J. Postgrad. Med..

[53] Shaffer J.A., Edmondson D., Taggart Wasson L., Falzon L., Homma K., Ezeokoli N., Li P., Davidson K.W. (2014). Vitamin D supplementation for depressive symptoms: A systematic review and meta-analysis of randomized controlled trials. Psychosom. Med..

[54] Spedding S. (2014). Vitamin D and depression: A systematic review and meta-analysis comparing studies with and without biological flaws. Nutrients.

[55] Cheng Y.C., Huang Y.C., Huang W.L. (2020). The effect of vitamin D supplement on negative emotions: A systematic review and meta-analysis. Depress. Anxiety.

[56] Hoffmann M.R., Senior P.A., Mager D.R. (2015). Vitamin D supplementation and health-related quality of life: A systematic review of the literature. J. Acad. Nutr. Diet..

[57] Farhangi M.A., Mesgari-Abbasi M., Nameni G., Hajiluian G., Shahabi P. (2017). The effects of vitamin D administration on brain inflammatory markers in high fat diet induced obese rats. BMC Neurosci..

[58] Walbert T., Jirikowski G.F., Prufer K. (2001). Distribution of 1,25-dihydroxyvitamin D3 receptor immunoreactivity in the limbic system of the rat. Horm. Metab. Res..

[59] Kim J.S., Kim Y.I., Song C., Yoon I., Park J.W., Choi Y.B., Kim H.T., Lee K.S. (2005). Association of vitamin D receptor gene polymorphism and Parkinson’s disease in Koreans. J. Korean Med. Sci..

[60] Perna S. (2019). Is vitamin D supplementation useful for weight loss programs? A systematic review and meta-analysis of randomized controlled trials. Medicina.

[61] Rondanelli M., Miccono A., Lamburghini S., Avanzato I., Riva A., Allegrini P., Faliva M.A., Peroni G., Nichetti M., Perna S. (2018). Self-Care for Common Colds: The Pivotal Role of Vitamin D, Vitamin C, Zinc, and Echinacea in Three Main Immune Interactive Clusters (Physical Barriers, Innate and Adaptive Immunity) Involved during an Episode of Common Colds-Practical Advice on Dosages and on the Time to Take These Nutrients/Botanicals in order to Prevent or Treat Common Colds. Evid. Based Complement. Alternat. Med..

