# Supplementary material for: Supplementation of Vitamin D and Mental Health in Adults with Respiratory System Diseases: A Systematic Review of Randomized Controlled Trials
Source: Nutrients. 2023 Feb 16;15(4):971. doi: 10.3390/nu15040971 (PMC9964312; doi:10.3390/nu15040971)
Supplement: Supplementary file 1 [file nutrients-15-00971-s001.zip › nutrients-2223422-supplementary.pdf]

Supplementary

Supplementation of Vitamin D and Mental Health in Adults with Respiratory System Diseases: A Systematic Review of Randomized Controlled Trials

Dominika Głąbska*, Aleksandra Kołota, Katarzyna Lachowicz, Dominika Skolmowska, Małgorzata Stachoń, and Dominika Guzek

Supplementary Table S1. The detailed electronic search strategy applied within the systematic review separately for PubMed and Web of Science databases.

Database	The applied full electronic search strategy
PubMed	(((mental health[T/A] OR mental disorders[T/A] OR mental disorder[T/A] OR psychological distress[T/A] OR mood disorders[T/A] OR depression[T/A] OR suicidal[T/A] OR suicide[T/A] OR anxiety[T/A] OR well-being[T/A] OR wellbeing[T/A] OR quality of life[T/A] OR self esteem[T/A] OR self-esteem[T/A] OR self efficacy[T/A] OR self-efficacy[T/A] OR resilience[T/A] OR empowerment[T/A] OR social participation[T/A] OR mental capital[T/A] OR life skills[T/A] OR emotional[T/A] OR psychology[T/A] OR psychosocial[T/A] OR psychiatry[T/A])) AND (vitamin D[T/A] OR vitamin D2[T/A] OR vitaminD3[T/A] OR D2[T/A] OR D3[T/A] OR ergocalciferol[T/A] OR cholecalciferol[T/A] OR 25-hydroxyvitamin D[T/A] OR 3-epi-25hydroxyvitaminD[T/A] OR calcitriol[T/A] OR dihydroxycholecalciferol[T/A])) NOT (animal NOT (animal AND human)[MeSH Terms])
Web of Science	(TS=("vitamin D" OR "vitamin D2" OR "vitamin D3" OR "D2" OR "D3" OR "ergocalciferol" OR "cholecalciferol" OR "25-hydroxyvitamin D" OR "3-epi-25 hydroxyvitamin D" OR "calcitriol" OR "dihydroxycholecalciferol") AND TS=("mental health" OR "mental disorders" OR "mental disorder" OR "psychological distress" OR "mood disorder" OR "depression" OR "suicidal" OR "suicide" OR "anxiety" OR "well-being" OR "wellbeing" OR "quality of life" OR "self esteem" OR "self-esteem" OR "self efficacy" OR "self-efficacy" OR "resilience" OR "empowerment" OR "social participation" OR "mental capital" OR "life skills" OR "emotional" OR "psychology" OR "psychosocial" OR "psychiatry") NOT TS=("animal" NOT ("animal" AND "human")))

T/A – Title/Abstract

Supplementary Table S2. Descriptions of the studies and studied populations within studies included in a systematic review.

Ref.	Observation	Conclusion
[28]	The quality of life did not significantly differ between the groups.	High-dose vitamin D supplementation in a sample of patients with COPD did not increase the quality of life.
[29]	The majority of patients, both placebo- and vitamin D treated, stated that they had felt 'better' during the study; 52% in the placebo group and 70% in the vitamin D group, relative risk 1.3 (95 % CI 1.0–1.8; $p = 0.06$, Fisher's exact test).	Vitamin D supplementation to patients with frequent RTIs might be beneficial, not only for infections, but also for their general well-being.
[30]	After 8 weeks, the treatment response or BDI scores did not differ significantly between groups. Multivariate logistic regression showed that BDI scores were not significantly improved in the Vit D group after adjustment for age, time to first negative smear, or 25-hydroxyvitamin D level.	The use of high-dose Vit D3 supplementation may not be warranted for reducing depressive symptoms in the PTB population.
[31]	In the intervention group, a significant difference was observed in quality of life at 2 months ($p < 0.001$) and 6 months ($p < 0.001$).	The current study shows that consumption of 50,000 IU vitamin D3, as a convenient supplementation in a daily diet, is able to increase quality of life in patients with COPD.
[32]	Supplemented patients showed improved quality of life compared with a placebo group, and the initial mini-AQLQ scores were improved for both groups.	In conclusion, in asthmatic patients with vitamin D deficiency, calcifediol supplementation compared with a placebo improved their quality of life.

COPD – Chronic Obstructive Pulmonary Disease; RTIs – Respiratory tract infections; BDI – Beck Depression Inventory; PTB – pulmonary tuberculosis; Mini-AQLQ – Mini Asthma Quality of Life Questionnaire.

Disclaimer/Publisher's Note: The statements, opinions and data contained in all publications are solely those of the individual author(s) and contributor(s) and not of MDPI and/or the editor(s). MDPI and/or the editor(s) disclaim responsibility for any injury to people or property resulting from any ideas, methods, instructions or products referred to in the content.
